# Emergence of a Carbapenem-Resistant *Klebsiella pneumoniae* Isolate Co-harbouring Dual *bla*_*NDM– 6*_-Carrying Plasmids in China

**DOI:** 10.3389/fmicb.2022.900831

**Published:** 2022-05-18

**Authors:** Yali Gong, Yifei Lu, Dongdong Xue, Yu Wei, Qimeng Li, Gang Li, Shuguang Lu, Jing Wang, Yunying Wang, Yizhi Peng, Yan Zhao

**Affiliations:** ^1^Institute of Burn Research, Southwest Hospital, State Key Lab of Trauma, Burn and Combined Injury, Chongqing Key Laboratory for Disease Proteomics, Army Medical University, Chongqing, China; ^2^Department of Radiology, Southwest Hospital, Army Medical University, Chongqing, China; ^3^Department of Microbiology, College of Basic Medical Sciences, Army Medical University, Chongqing, China; ^4^Department of Laboratory Medicine, The Second Affiliated Hospital, Chongqing Medical University, Chongqing, China

**Keywords:** *Klebsiella pneumoniae*, carbapenem resistance, IncN, complete genome sequence, *bla*_*NDM–6*_

## Abstract

The widespread emergence of carbapenem-resistant *Klebsiella pneumoniae* (CRKP) with limited therapeutic options has become a global concern. In this study, a *K. pneumoniae* strain called KP2e was recovered from a human case of fatal septic shock in a Chinese hospital. Polymerase chain reaction and sequencing, antimicrobial susceptibility testing, conjugation experiments, S1 nuclease-pulsed field gel electrophoresis/southern blot, whole genome sequencing and comparative genomics were performed to investigate the phenotypic and molecular characteristics of this isolate. KP2e possessed the NDM-6-encoding gene and exhibited resistance to almost all β-lactams except for monobactam. This strain belonged to sequence type 4024, the complete genome of which was composed of one chromosome and three plasmids. Furthermore, *bla*_*NDM–6*_ coexisted on two self-transmissible plasmids, which were assigned to types IncFIB and IncN. A structure of IS*26*-composite transposon capturing an identical Tn*125* remnant (ΔIS*Aba125*-*bla*_*NDM–6*_-*ble*_*MBL*_-*trpF*-*dsbC*-*cutA*-*groES*-Δ*groEL*) was identified in the two plasmids, and this conserved *bla*_*NDM*_-surrounding genetic context was similar to that of few IncN plasmids found in other regions of China. Our research appears to be the first description of a clinical strain that emerged co-harbouring dual *bla*_*NDM*_-carrying plasmids, and the first report of NDM-6-positive CRKP in China. These findings demonstrated that IncN is a key medium in the evolution and expanding dissemination of *bla*_*NDM*_ genes among various species, which indicates that close monitoring and rapid detection of *bla*_*NDM*_-harbouring plasmids is necessary.

## Introduction

The prevalence of carbapenem-resistant Enterobacterales (CRE), which has evolved with critical resistance to first-line antibiotics, represents a severe challenge for clinical therapy and is an urgent threat to the global healthcare setting (Zhang et al., 2017; Nordmann and Poirel, 2019; Corcione et al., 2020; De Oliveira et al., 2020). Carbapenem-resistant *Klebsiella pneumoniae* (CRKP) isolates often cause serious infections with a high mortality rate and are, thus, the most clinically prominent CRE (Greenhalgh, 2017; Theuretzbacher et al., 2020; Yang et al., 2021). Carbapenem resistance in *K. pneumoniae* is usually dominated by acquisition and high diversity of plasmid-encoding carbapenemase enzymes, such as *K. pneumoniae* carbapenemases (KPCs), extended-spectrum β-lactamases (ESBLs), and MBLs (metallo-β-lactamases) (Paczosa and Mecsas, 2016; Navon-Venezia et al., 2017). MBL-positive *K. pneumoniae* strains, especially the newly emerging NDM type, are the most critical-priority pathogens due to their high variability, tachytelic evolution, and global dissemination (Wu et al., 2019; Bush and Bradford, 2020).

Since the original detection of NDM-1 in *K. pneumoniae* isolated from a Swedish patient, who had received healthcare in New Delhi, India, in 2008, 33 variants of NDM have been identified across all continents (Yong et al., 2009; Wang et al., 2021). NDM-6 is different from the NDM-1 allele due to a single amino acid substitution (A233V), which resulted in enhanced stability of the enzyme against proteolytic degradation (Bahr et al., 2018; Mehaffey et al., 2020). To date, only sporadic studies focusing on NDM-6-producing strains have been reported, with *Escherichia coli*, *Acinetobacter baumannii*, and *K. pneumoniae* reported in New Zealand, Spain and Iran, respectively (Williamson et al., 2012; Bahramian et al., 2019; Xanthopoulou et al., 2020). In addition, a clinical isolate producing NDM-6 has not been described in China.

In this study, we identified a clinical NDM-6-producing *K. pneumoniae* isolate that was recovered from an electrically injured patient, and characterised its phenotypic and molecular profiles. Rarely, plasmid detection and analyses demonstrate that this isolate simultaneously co-harbours two different types of plasmids encoding NDM-6. Accordingly, our investigation seems to first report the emergence of the NDM-6-positive *K. pneumoniae* strain in China and contributes to elucidating the genetic contexts that mediate the potential transfer mechanisms of the *bla*_*NDM–6*_ gene.

## Materials and Methods

### Bacterial Strains and Species Identification

Since July 2011, various clinical strains were recovered from burn patients who were hospitalised in the Institute of Burn Research, First Affiliated Hospital of Army Medical University (Chongqing, China). An NDM-producing isolate, *K. pneumoniae* strain 2e (KP2e), was collected from the blood sample of a patient with high-voltage electric injury involving 75% of the total body-surface area in August 2017. Disc diffusion assays (for imipenem and meropenem) were used to obtain carbapenem-resistant isolates. Species identification was performed utilising both 16S rRNA gene sequencing and the VITEK-2 compact system (bioMérieux, Lyon, France) following the manufacturer’s instructions. The carbapenemase activity was tested *via* the modified carbapenem inactivation method (mCIM) and EDTA-modified carbapenem inactivation method (eCIM) (CLSI, 2018). Polymerase chain reactions (PCRs) for the *bla*_*NDM*_, *bla*_*OXA–48*_, *bla*_*KPC*_, *bla*_*IMP*_, and *bla*_*VIM*_ genes were conducted to investigate the carbapenemase genes. Primers used in this experiment are listed in Supplementary Table 1. The positive DNA fragments were purified using Wizard^®^ SV Gel and the PCR Clean-Up System (Promega, Madison, WI, United States), then sequenced by the Beijing Genomics Institute (BGI, Beijing, China).

### Conjugal Transfer of Plasmids and Detection of the *bla*_*NDM–6*_ Gene

As described previously (Wailan et al., 2015), filter-mating conjugation experiments were carried out using KP2e as the donor and azide-resistant *E. coli* J53 as the recipient. Transconjugants that possessed the *bla*_*NDM–6*_-carrying plasmid were detected on Mueller–Hinton agar plates (Oxoid, Hampshire, United Kingdom) containing 1 mg/L imipenem plus 100 mg/L sodium azide. The number and sizes of plasmid-located *bla*_*NDM–6*_ were estimated by S1 nuclease-pulsed field gel electrophoresis (S1-PFGE) followed by southern blot (Chen et al., 2015). Briefly, the linearised plasmids digested by S1 nuclease (Takara, Shiga, Japan) were separated *via* the CHEF Mapper XA system (Bio-Rad, Hercules, CA, United States). Furthermore, the DNA fragments were transferred to a positively charged nylon membrane (Millipore, United States) and then hybridised with a digoxigenin (DIG)-labelled probe (Roche Diagnostics, Basel, Switzerland) specific against *bla*_*NDM*_. The signal was detected with a DIG Luminescent Detection Kit (Sigma-Aldrich, MO, United States) and visualised through a Fusion Pulse 6 imaging system (Vilber, Marne-la-Vallée, France).

### Antimicrobial Susceptibility Testing

The VITEK-2 compact system and agar dilution method were employed here to assess the antimicrobial susceptibility profiles of the KP2e isolate and corresponding transconjugants. *E. coli* ATCC 25922 was used as a quality control strain. A total of 19 antibiotics, including ampicillin, ampicillin/sulbactam, piperacillin/tazobactam, aztreonam, cefazolin, cefotetan, ceftriaxone, ceftazidime, cefepime, imipenem, ertapenem, ciprofloxacin, levofloxacin, amikacin, gentamicin, tobramycin, tigecycline, polymyxin B, and trimethoprim/sulfamethoxazole, were tested. Minimum inhibitory concentrations (MICs) were interpreted according to the Clinical and Laboratory Standards Institute (CLSI) document M100-S28 (CLSI, 2018), except for tigecycline and polymyxin B, for which results were judged in line with the European Committee on Antimicrobial Susceptibility Testing guidelines (EUCAST, 2016).

### Whole Genome Sequencing, Assembly, Annotation, and Bioinformatics Analysis

The total genomic DNA of KP2e was extracted by E.Z.N.A.^®^ Bacterial DNA Kit (Omega Bio-tek, Atlanta, GA, United States). For the complete sequences of genome and plasmids, DNA subjected to whole genome sequencing (WGS) was sequenced on HiSeq 4000-PE150 (Illumina, San Diego, CA, United States) combined with the PacBio RSII platform (Pacific Biosciences, Menlo Park, CA, United States). The genomic DNA library with 250-bp paired-end reads was prepared using Illumina^®^ DNA Prep Kits (Illumina^®^, San Diego, CA, United States). Utilising Unicycler,^1^ Illumina sequence reads were then *de novo* assembled in contigs through SPAdes.^2^ For PacBio RSII, the SMRTbell template libraries were prepared using g-TUBE (Covaris, Woburn, MA, United States) and SMRTbell adapters (Pacific Biosciences, Menlo Park, CA, United States). By using a semi-global alignment algorithm, Unicycles implemented PacBio long reads into the assembly graph, and Illumina sequencing reads were mapped back onto the sequence generated from PacBio data to validate the genome assembly (Wick et al., 2017). RAST^3^ was applied to add the annotation and predict open reading frames (ORFs) (Aziz et al., 2008). Gene structures were visualised and analysed using SnapGene V 4.1.8.

Multilocus sequence typing (MLST) was determined by the *K. pneumoniae* MLST website.^4^ Additionally, the acquired antimicrobial resistance genes and plasmid replicon typing were manually identified through a direct search against the database of the Center for Genomic Epidemiology.^5^ Identification of transposons and insertion sequences (IS) elements was performed using ISfinder^6^ (Siguier et al., 2006). Finally, the BLAST Ring Image Generator (BRIG) was employed to perform multiple comparisons of complete plasmid sequences available at the National Center for Biotechnology Information (NCBI), and circular maps were generated (Alikhan et al., 2011). A linear comparison of the genetic context surrounding *bla*_*NDM–6*_ was performed using BLASTn, and the image was fabricated by Easyfig^7^ (Sullivan et al., 2011).

### Nucleotide Sequence Accession Numbers

The complete nucleotide sequences of each the chromosome and three plasmids of KP2e were deposited in GenBank under accession numbers CP040175, CP040176, CP040177, and CP040178, respectively.

## Results

### Screening and Identification of the NDM-6-Producing *Klebsiella pneumoniae* Strain

In August 2017, a 15-year-old patient, who was initially diagnosed with high-voltage electric injury involving 75% of the total body-surface area, was admitted to the Burn Intensive Care Unit of the Institute of Burn Research. This patient underwent femoral artery catheterisation, debridement, autogenous mesh skin grafting and antimicrobial treatment during the period of hospitalisation. However, typically symptoms of systemic infection developed soon after surgery and, finally, the patient died of septic shock in September 2017. On the 21st day post admission, a Gram-negative bacteria with a rod shape was detected from the blood specimens, and carbapenem resistance of the isolate was observed *via* cultivation in selective medium (imipenem and meropenem). This clinical isolate designated 2e was identified as *K. pneumoniae* by 16S rRNA gene sequencing and the VITEK-2 compact system. KP2e, which exhibited carbapenemase activity in the mCIM and eCIM assays, was confirmed to carry the *bla*_*NDM*_ gene after multiplex PCR. Then, the *bla*_*NDM–6*_ gene was determined *via* amplicon sequencing.

### Antimicrobial Susceptibility Testing

On the basis of the VITEK2-compact system and agar dilution method, the detected antimicrobial susceptibility phenotype of KP2e is listed in Table 1. KP2e showed resistance to almost all β-lactams tested, including ampicillin (MIC ≥ 32 μg/ml), ampicillin/sulbactam (MIC ≥ 32 μg/ml), piperacillin/tazobactam (MIC ≥ 128 μg/ml), cefazolin (MIC ≥ 64 μg/ml), cefotetan (MIC ≥ 64 μg/ml), ceftriaxone (MIC ≥ 64 μg/ml), ceftazidime (MIC ≥ 64 μg/ml), cefepime (MIC ≥ 64 μg/ml), imipenem (MIC ≥ 16 μg/ml), and ertapenem (MIC ≥ 8 μg/ml), except for aztreonam (MIC = 2 μg/ml). This isolate was also resistant to ciprofloxacin (MIC = 2 μg/ml) and trimethoprim/sulfamethoxazole (MIC ≥ 320 μg/ml) and demonstrated intermediate resistant to levofloxacin (MIC = 1 μg/ml) and tobramycin (MIC = 8 μg/ml). Nevertheless, KP2e exhibited susceptibility to amikacin (MIC ≤ 2 μg/ml) and with an MIC ≤ 1 μg/ml for gentamicin, tigecycline, and polymyxin B.

**TABLE 1:** Minimum inhibitory concentration values of antimicrobials for KP2e, recipient strain *E. coli* J53 and transconjugant KP2e-J53.

Category	Antibiotics	MIC (μg/ml)/		
		antimicrobial susceptibility		
		KP2e	KP2e-J53	J53
Penicillin	Ampicillin	≥32/R	≥32/R	8/S
	Ampicillin/sulbactam	≥32/R	≥32/R	4/S
	Piperacillin/tazobactam	≥128/R	≥128/R	≤4/S
Monobactam	Aztreonam	2/S	≤1/S	≤1/S
Cephalosporin	Cefazolin	≥64/R	≥64/R	≤4/S
	Cefotetan	≥64/R	≥64/R	≤4/S
	Ceftriaxone	≥64/R	≥64/R	≤1/S
	Ceftazidime	≥64/R	≥64/R	≤1/S
	Cefepime	≥64/R	≥64/R	≤1/S
Carbapenem	Imipenem	≥16/R	≥16/R	≤1/S
	Ertapenem	≥8/R	≥8/R	≤0.5/S
Fluoroquinolone	Ciprofloxacin	2/R	1/R	≤0.25/S
	Levofloxacin	1/I	1/I	≤0.25/S
Aminoglycoside	Amikacin	≤2/S	≤2/S	≤2/S
	Gentamicin	≤1/S	≤1/S	≤1/S
	Tobramycin	8/I	8/I	≤1/S
Tetracycline	Tigecycline	1/S	1/S	1/S
Polymyxins	Polymyxin B	1/S	1/S	1/S
Sulphanilamide	Trimethoprim/sulfamethoxazole	≥320/R	≥320/R	≤20/S

*S, sensitive; R, resistant; I, intermediate.*

### Genotypic Characterisation of the KP2e Strain

*In silico* analysis of the generally molecular characteristics of the KP2e strain are tabulated in Table 2. The results of WGS demonstrated that KP2e possessed a 5,208,029-bp circular chromosome with 57.61% guanine-cytosine (G + C) content and three plasmids of 247,125, 28,566, and 59,952 bp in size. According to the RAST analysis, there were 5,008 putative ORFs and 112 RNA genes on the circular chromosome. The MLST result indicated that KP2e belonged to ST4024, and there was no recorded ST4024 strain in the Pasteur database of *K. pneumoniae*. The replicon type of the three plasmids were assigned to IncFIB, IncR and IncN, respectively. A search with ResFinder identified 14 acquired resistance genes located in KP2e, including the *bla*_*CTX–M–14*_ gene encoding extended-spectrum β-lactamase (ESBL) in each the chromosome and plasmid 1, *bla*_*SHV–182*_ encoding class A β-lactamase in the chromosome, and *bla*_*NDM–6*_ in each plasmid 1 and plasmid 3. In addition, the genes *fosA*, *OqxA*, and *OqxB* were also identified in the chromosome, for which *sul1*, *dfrA1*, *tet(A)*, and *qacE* in plasmid 1; *mph(A)* and *aac(6′)-1b-cr* in plasmid 2; and *qnrs1*, *dfrA14* in plasmid 3 (Table 2). These genes mediate resistance to a diverse array of antibiotics that correspond to the drug susceptibility spectrum.

**TABLE 2:** Genomic features of KP2e.

Feature	Chromosome	Plasmid 1	Plasmid 2	Plasmid 3
Total number of bases (bp)	5208029	247125	28566	59952
G + C content (%)	57.61	51.83	54.87	52.41
Circular	Yes	Yes	No	Yes
Number of open reading frames	5008	338	49	94
Number of RNAs	112	0	0	0
Plasmid type	–	IncFIB	IncR	IncN
Resistance genes	*bla*_*CTX–M–14*_, *bla*_*SHV–182*_, *fosA*, *OqxA*, *OqxB*	*bla*_*CTX–M–14*_, *bla*_*NDM–6*_, *sul1*, *dfrA1*, *tet(A)*, *qacE*	*mph(A), aac(6′)-1b-cr*	*bla*_*NDM–6*_, *qnrS1*, *dfrA14*
Accession numbers	CP040175	CP040176	CP040177	CP040178

### Characterisation and Comparative Genomics of Plasmids Carrying *bla*_*NDM–6*_

**FIGURE 1:**
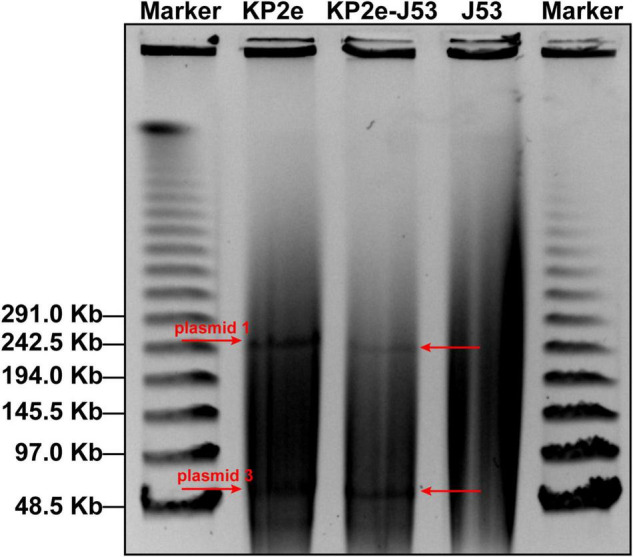
Determination of *bla*_*NDM–6*_-carrying plasmids by S1-PFGE/southern blot. The S1-digested genomic DNA samples were analysed on the PFGE gel, then subjected to southern blot hybridisation with a DIG-labelled probe specific to *bla*_*NDM*_.

The S1-PFGE/southern hybridisation result demonstrated that the *bla*_*NDM–6*_ gene was localised in plasmids 1 and 3, both of which could be horizontally transferred from the donor KP2e to the recipient *E. coli* J53 (Figure 1). Compared to the wild type, the antimicrobial resistance of this transconjugant was completely identical to that of KP2e (Table 1). Hence, these self-transmissible plasmids mainly account for the acquirement of *bla*_*NDM–6*_-derived carbapenem resistance.

**FIGURE 2:**
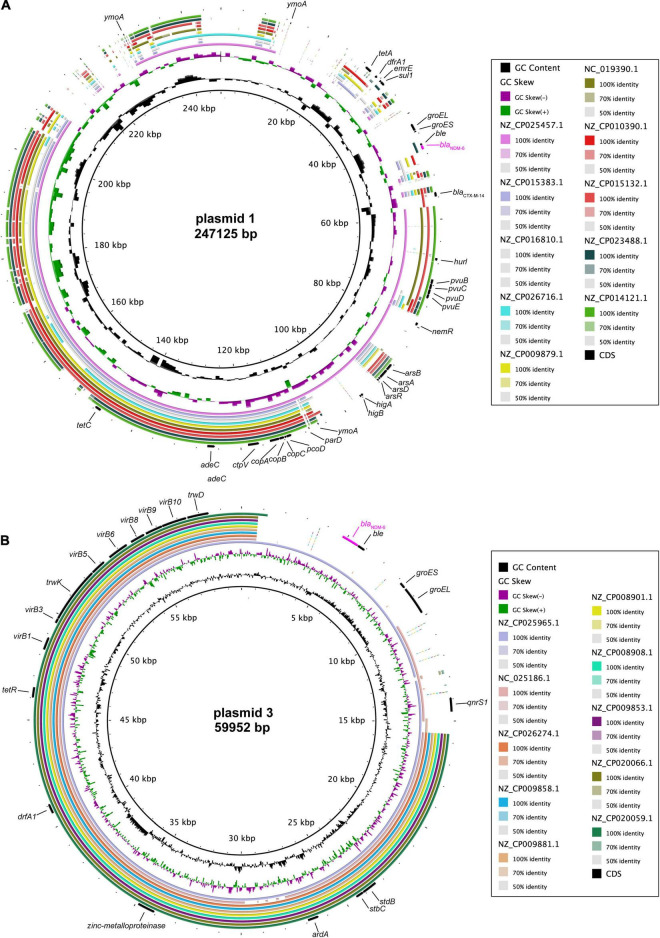
Comparative genomics of *bla*_*NDM–6*_-harbouring plasmids. Comparison of plasmid 1 **(A)** and plasmid 3 **(B)** with closely related plasmids deposited in GenBank. Concentric rings represent the similarity between the reference sequence in the inner ring and other sequences in the outer rings. Colour levels indicate the result of BLASTn with a matched degree in the shared regions.

Complete DNA sequencing showed that *bla*_*NDM–6*_-harbouring plasmid 1, with a G + C content of 51.83%, carries a total of 338 ORFs (Table 2), and multiple alignments of complete plasmid sequences were performed by comparing plasmid 1 with similar plasmids available at GenBank, including plasmid p69-1 (*K. pneumoniae*, China, no. NZ_CP025457.1), pCN1_1 (*K. pneumoniae*, China, no. NZ_CP015383.1), and p_IncFIB_DHQP1002001 (*K. pneumoniae*, United States, no. NZ_CP016810.1). Thereinto, IncFIB-type plasmid p69-1 shared the greatest similarity to plasmid 1 in the backbone composition but varied in the large stretches of regions containing antimicrobial resistance genes, including *bla*_*NDM–6*_ (Figure 2A). Meanwhile, plasmid 3, assigned to the IncN replicon type, contained 94 annotated ORFs with an average G + C content of 52.41% (Table 2). A multiple comparison result demonstrated the most sequence identity of 99% and coverages of 100% with one *bla*_*NDM–1*_-carrying plasmid pNDM1_LL34 (*K. pneumoniae*, China, no. NZ_CP025965.1) (Figure 2B; Liu et al., 2018). These results indicated that the IncFIB-type plasmid 1 was more likely to be a novel vector integrated with the *bla*_*NDM–6*_-carrying accessory module, whereas IncN-type plasmid 3 was acquired through horizontal transfer.

**FIGURE 3:**
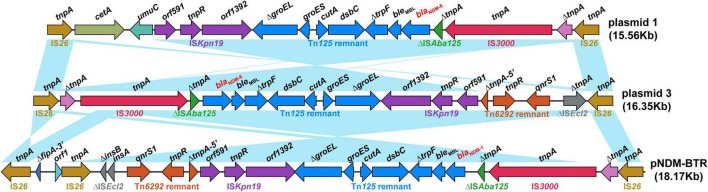
Structure of the *bla*_*NDM–6*_ region and flanking mobile elements. Linear genomic comparison between plasmid 1, plasmid 3, and pNDM-BTR (as a reference) of the conserved genetic context containing *bla*_*NDM*_. Open reading frames are denoted by arrows. Genes, mobile elements and other features are coloured based on their functional classification. Blue shading regions represent regions of homology (nucleotide identity, >95%).

As shown in Figure 3, Blastn, a conserved structure of gene context surrounding *bla*_*NDM–6*_ termed as IS*26*-formed composite transposon, was identified in plasmids 1 and 3, and were arranged in the following order: IS*26*, truncated IS*3*, IS*3000*, truncated IS*Aba125*, *bla*_*NDM–6*_, *ble*_*MBL*_ (resistance to bleomycin), *trpF* (phosphoribosylanthranilate isomerase), *dsbC* (twin-arginine translocation pathway signal sequence domain protein), *cutA* (divalent-cation tolerance protein), *groES* (co-chaperone GroES), truncated *groEL* (chaperonin GroEL), IS*Kpn19*, and IS*26*. Furthermore, a linear genomic comparison of the conserved region was performed between the two plasmids and a previously reported IncN-type plasmid pNDM-BTR (*bla*_*NDM–1*_-carrying, *E. coli*, China, no. KF534788.1.) (Zhao et al., 2017), which shared a sequence identity of 99.78% and coverage of 100%. Compared to plasmid 1 and pNDM-BTR, the genetic context containing *bla*_*NDM*_ was inverted in plasmid 3. Inside the two opposite-orientated IS*26* elements, a IS*3000*-formed transposon (termed Tn*3000*) was truncated by IS*Kpn19* at one extremity. Meanwhile, the Tn*125* remnant (ΔIS*Aba125*-*bla*_*NDM–6*_-*ble*_*MBL*_-*trpF*-*dsbC*-*cutA*-*groES*-Δ*groEL*), which was aborted by deleting the original IS*CR27* and second IS*Aba125*, was inserted into a site downstream of the IS*3000*. However, a Tn*6292* remnant encompassing *qnrs1* (resistance to quinolone) was found in plasmid 3 and pNDM-BTR but replaced by *umuC* (DNA polymerase V subunit UmuC) and *cetA* (HAMP domain-containing protein) in plasmid 1. These characteristics suggested that insertion sequence IS*26* was responsible for intermolecular transposition along the backbone of plasmids, and *bla*_*NDM–6*_-flanking mobile elements, such as IS*3000*, IS*Kpn19*, IS*Aba125*, and Tn*125*, played an important role in multiple recombination events and wide dissemination among bacterial species.

## Discussion

The present study characterises a new CRKP isolated from a clinical blood specimen, which was identified to possess dual *bla*_*NDM–6*_-carrying plasmids of distinct incompatibility groups. Since NDM-1 has been found widely in a total of 11 bacterial host families, new variants of NDM containing between 1 and 5 amino acid alterations have been continually generated, which indicates that amino acid substitution dominates the extension of MBL activity (Wu et al., 2019). A233V substitution enabled NDM-6 to evolve higher enzyme fitness in the periplasm under the Zn(II) starvation condition than NDM-1 (Bahr et al., 2018; Sychantha et al., 2021). To date, there are four additional *bla*_*NDM–6*_-harbouring plasmids with complete sequences deposited in NCBI, which belonged to incompatibility groups IncHI2, IncX3, IncFII, and IncR from *Leclercia adecarboxylata*, *K. pneumoniae* and *E. coli.* The success of conjugal transfer of IncFIB- and IncN-type plasmids from clinical strain KP2e to *E. coli* J53, thereby conferring resistance against carbapenem, highlighted that multiple plasmids are the major source of acquisition of *bla*_*NDM–6*_.

IncFIB-type plasmid 1 exhibited low sequence identity with plasmids available at GenBank, whereas plasmid 3 was highly similar to another NDM-1-encoding plasmid, pNDM1_LL34, which also belonged to the IncN group of a *K. pneumoniae* strain isolated from a 72-year-old male patient with pancreatitis in Chengdu, China, in 2017 (Liu et al., 2018). IncN-type plasmids have been identified to frequently colocalise with IncF-type plasmids, and a fusion between the two groups of plasmids has been reported (Yang et al., 2014; Rozwandowicz et al., 2018). Based on these factors, we suppose that a common IncN plasmid mediated interspecies dissemination and an integrative recombination of independent plasmids occurred within KP2e. It is evident that IncF-type plasmids are the most frequently described vehicles involved in mediating *bla*_*NDM*_ spread among Enterobacteriaceae from multiple continents (Zou et al., 2020; Zhang et al., 2021).

On plasmids 1 and 3, a conserved genetic context flanking *bla*_*NDM–6*_ was bracketed by two copies of IS*26* elements, which showed high identity with a well-characterised plasmid pNDM-BTR from an NDM-1-positive *E. coli* strain isolated in Beijing, China, in 2013. Recently, a similar IS*26*-formed composite transposon comprising *bla*_*NDM*_ was observed in a new NDM-29 (G388A)-encoding plasmid, pNC225-NDM-29, which was also assigned to the IncN group and found in *E. coli* in Jiangxi, China, in 2019 (Zhu et al., 2021). Considering the reports of these IS*26* dominating accessory modules around China and constant emergence of NDM variants derived from mutations (Zhao et al., 2021), we deduced that IS*26* dramatically contributes to the transmission and evolution of the *bla*_*NDM*_ gene. Tn*125*, which is formed by IS*Aba125*, is the most common transposable element involved in the interspecies transmission of *bla*_*NDM*_ among *Acinetobacter* spp. and Enterobacteriaceae (Bontron et al., 2016; Zafer et al., 2021). IS*Aba125* in the upstream (intact or interrupt) provides a strong promoter for driving the expression of *bla*_*NDM*_ (Poirel et al., 2011; Toleman et al., 2012). However, the second IS*Aba125* of Tn*125* is truncated by IS*Kpn19*, and in plasmid 1, the downstream Tn*6292* containing a core *qnrS1* genetic platform was obliterated, which indicated that IS*Kpn19* is associated with intermolecular integrative transposition between distinct plasmids (Gauthier et al., 2019). In addition, the presence of IS*3000* and other various mobile elements capturing *bla*_*NDM*_-carrying Tn*125* also indicates that multiple recombination events and complex mechanisms accompany transfer resistance genes (Campos et al., 2015; Chen et al., 2020a,b).

In conclusion, we first report an NDM-6-producing *K. pneumoniae* isolate that caused a fatal septic shock in China. A total of 14 resistance genes were identified in this strain, and the *bla*_*NDM–6*_ gene was simultaneously located on two self-transmissible plasmids of IncFIB and IncN groups within a clinical strain. Further exploration of factors that facilitate KP2e evolution to obtain multiple resistance genes against the same class of antibiotics is needed. These phenotypic and genetic characterisations provide a deeper insight into the molecular mechanisms of horizontal transfer and evolution of the *bla*_*NDM*_ gene. Furthermore, this study highlights the important role of the IncN plasmid in mediating wide dissemination and evolution of NDM in China. Hence, epidemiological investigations and close surveillance must be implemented to prevent and reduce the prevalence of NDM variants.

## Data Availability Statement

The datasets presented in this study can be found in online repositories. The names of the repository/repositories and accession number(s) can be found in the article/Supplementary Material.

## Author Contributions

YP and YZ conceived the study. YG and YL performed the experiments and bioinformatics analysis. DX, YWe, QL, GL, SL, JW, and YW analysed and interpreted the data. YL and YZ wrote the manuscript. All authors checked the manuscript and submitted the final version.

## Conflict of Interest

The authors declare that the research was conducted in the absence of any commercial or financial relationships that could be construed as a potential conflict of interest.

## Publisher’s Note

All claims expressed in this article are solely those of the authors and do not necessarily represent those of their affiliated organizations, or those of the publisher, the editors and the reviewers. Any product that may be evaluated in this article, or claim that may be made by its manufacturer, is not guaranteed or endorsed by the publisher.

## Footnotes

1
github.com/rrwick/Unicycler

2
github.com/ablab/spades

3
rast.nmpdr.org

4
https://bigsdb.pasteur.fr/klebsiella

5
http://www.genomicepidemiology.org/

6
http://www-is.biotoul.fr/

7
https://github.com/mjsull/Easyfig

## References

[B1] Alikhan N. F. Petty N. K. Ben Zakour N. L. Beatson S. A. (2011). BLAST Ring Image Generator (BRIG): simple prokaryote genome comparisons. *BMC Genomics* 12:402. 10.1186/1471-2164-12-402 21824423 PMC3163573

[B2] Aziz R. K. Bartels D. Best A. A. Dejongh M. Disz T. Edwards R. A. (2008). The RAST Server: rapid annotations using subsystems technology. *BMC Genomics* 9:75. 10.1186/1471-2164-9-75 18261238 PMC2265698

[B3] Bahr G. Vitor-Horen L. Bethel C. R. Bonomo R. A. González L. J. Vila A. J. (2018). Clinical evolution of new delhi metallo-β-lactamase (NDM) optimizes resistance under Zn(II) deprivation. *Antimicrob. Agents Chemother.* 62:e01849–17. 10.1128/aac.01849-17 29038264 PMC5740384

[B4] Bahramian A. Shariati A. Azimi T. Sharahi J. Y. Bostanghadiri N. Gachkar L. (2019). First report of new delhi metallo-β-lactamase-6 (NDM-6) among *Klebsiella pneumoniae* ST147 strains isolated from dialysis patients in Iran. *Infect. Genet. Evol.* 69 142–145. 10.1016/j.meegid.2019.01.030 30684646

[B5] Bontron S. Nordmann P. Poirel L. (2016). Transposition of Tn125 encoding the NDM-1 carbapenemase in *Acinetobacter baumannii*. *Antimicrob. Agents Chemother.* 60 7245–7251. 10.1128/aac.01755-16 27671058 PMC5119004

[B6] Bush K. Bradford P. A. (2020). Epidemiology of β-lactamase-producing pathogens. *Clin. Microbiol. Rev.* 33:e00047–19. 10.1128/cmr.00047-19 32102899 PMC7048014

[B7] Campos J. C. Da Silva M. J. Dos Santos P. R. Barros E. M. Pereira Mde O. Seco B. M. (2015). Characterization of Tn3000, a transposon responsible for blaNDM-1 dissemination among *Enterobacteriaceae* in Brazil, Nepal, Morocco, and India. *Antimicrob. Agents Chemother.* 59 7387–7395. 10.1128/aac.01458-15 26392506 PMC4649174

[B8] Chen Q. Lin Y. Li Z. Lu L. Li P. Wang K. (2020a). Characterization of a new transposon, Tn6696, on a blaNDM-1-carrying plasmid from multidrug-resistant *Enterobacter cloacae* ssp. dissolvens in China. *Front. Microbiol.* 11:525479. 10.3389/fmicb.2020.525479 33042048 PMC7522282

[B9] Chen Q. Zhou J. Wu S. Yang Y. Yu D. Wang X. (2020b). Characterization of the IncX3 plasmid producing blaNDM-7 from *Klebsiella pneumoniae* ST34. *Front. Microbiol.* 11:1885. 10.3389/fmicb.2020.01885 32849464 PMC7419432

[B10] Chen Z. Li H. Feng J. Li Y. Chen X. Guo X. (2015). NDM-1 encoded by a pNDM-BJ01-like plasmid p3SP-NDM in clinical *Enterobacter* aerogenes. *Front. Microbiol.* 6:294. 10.3389/fmicb.2015.00294 25926823 PMC4396501

[B11] CLSI (2018). *Performance Standards for Antimicrobial Susceptibility Testing*, 28th Edn. Wayne, PA: Clinical and Laboratory Standards Institute.

[B12] Corcione S. Lupia T. De Rosa F. G. (2020). Microbiome in the setting of burn patients: implications for infections and clinical outcomes. *Burns Trauma* 8:tkaa033. 10.1093/burnst/tkaa033 32821744 PMC7428410

[B13] De Oliveira D. M. P. Forde B. M. Kidd T. J. Harris P. N. A. Schembri M. A. Beatson S. A. (2020). Antimicrobial resistance in ESKAPE pathogens. *Clin. Microbiol. Rev.* 33:e00181–19. 10.1128/cmr.00181-19 32404435 PMC7227449

[B14] EUCAST (2016). *Breakpoint Tables for Interpretation of MICs and Zone Diameters, Version 6.0.* Växjö: EUCAST.

[B15] Gauthier L. Dortet L. Jousset A. B. Mihaila L. Golse N. Naas T. (2019). Molecular characterization of plasmid-encoded Tripoli MBL 1 (TMB-1) in *Enterobacteriaceae*. *J. Antimicrob. Chemother.* 74 42–47. 10.1093/jac/dky372 30252055

[B16] Greenhalgh D. G. (2017). Sepsis in the burn patient: a different problem than sepsis in the general population. *Burns Trauma* 5:23. 10.1186/s41038-017-0089-5 28795054 PMC5547526

[B17] Liu L. Feng Y. Long H. Mcnally A. Zong Z. (2018). Sequence Type 273 carbapenem-resistant *Klebsiella pneumoniae* carrying blaNDM-1 and blaIMP-4. *Antimicrob. Agents Chemother.* 62:e00160–18. 10.1128/aac.00160-18 29610206 PMC5971603

[B18] Mehaffey M. R. Ahn Y. C. Rivera D. D. Thomas P. W. Cheng Z. Crowder M. W. (2020). Elusive structural changes of New Delhi metallo-β-lactamase revealed by ultraviolet photodissociation mass spectrometry. *Chem. Sci.* 11 8999–9010. 10.1039/d0sc02503h 34123154 PMC8163344

[B19] Navon-Venezia S. Kondratyeva K. Carattoli A. (2017). *Klebsiella pneumoniae*: a major worldwide source and shuttle for antibiotic resistance. *FEMS Microbiol. Rev.* 41 252–275. 10.1093/femsre/fux013 28521338

[B20] Nordmann P. Poirel L. (2019). Epidemiology and diagnostics of carbapenem resistance in gram-negative bacteria. *Clin. Infect. Dis.* 69 S521–S528. 10.1093/cid/ciz824 31724045 PMC6853758

[B21] Paczosa M. K. Mecsas J. (2016). *Klebsiella pneumoniae*: going on the offense with a strong defense. *Microbiol. Mol. Biol. Rev.* 80 629–661. 10.1128/mmbr.00078-15 27307579 PMC4981674

[B22] Poirel L. Dortet L. Bernabeu S. Nordmann P. (2011). Genetic features of blaNDM-1-positive *Enterobacteriaceae*. *Antimicrob. Agents Chemother.* 55 5403–5407. 10.1128/aac.00585-11 21859933 PMC3195013

[B23] Rozwandowicz M. Brouwer M. S. M. Fischer J. Wagenaar J. A. Gonzalez-Zorn B. Guerra B. (2018). Plasmids carrying antimicrobial resistance genes in *Enterobacteriaceae*. *J. Antimicrob. Chemother.* 73 1121–1137. 10.1093/jac/dkx488 29370371

[B24] Siguier P. Perochon J. Lestrade L. Mahillon J. Chandler M. (2006). ISfinder: the reference centre for bacterial insertion sequences. *Nucleic Acids Res.* 34 D32–D36. 10.1093/nar/gkj014 16381877 PMC1347377

[B25] Sullivan M. J. Petty N. K. Beatson S. A. (2011). Easyfig: a genome comparison visualizer. *Bioinformatics* 27 1009–1010. 10.1093/bioinformatics/btr039 21278367 PMC3065679

[B26] Sychantha D. Rotondo C. M. Tehrani K. Martin N. I. Wright G. D. (2021). Aspergillomarasmine A inhibits metallo-β-lactamases by selectively sequestering Zn(2). *J. Biol. Chem.* 297:100918. 10.1016/j.jbc.2021.100918 34181945 PMC8319579

[B27] Theuretzbacher U. Bush K. Harbarth S. Paul M. Rex J. H. Tacconelli E. (2020). Critical analysis of antibacterial agents in clinical development. *Nat. Rev. Microbiol.* 18 286–298. 10.1038/s41579-020-0340-0 32152509

[B28] Toleman M. A. Spencer J. Jones L. Walsh T. R. (2012). blaNDM-1 is a chimera likely constructed in *Acinetobacter baumannii*. *Antimicrob. Agents Chemother.* 56 2773–2776. 10.1128/aac.06297-11 22314529 PMC3346620

[B29] Wailan A. M. Sartor A. L. Zowawi H. M. Perry J. D. Paterson D. L. Sidjabat H. E. (2015). Genetic contexts of blaNDM-1 in patients carrying multiple NDM-producing strains. *Antimicrob. Agents Chemother.* 59 7405–7410. 10.1128/aac.01319-15 26392493 PMC4649221

[B30] Wang T. Zhou Y. Zou C. Zhu Z. Zhu J. Lv J. (2021). Identification of a novel blaNDM variant, blaNDM-33 in an *Escherichia coli* isolate from hospital wastewater in china. *mSphere* 6:e0077621. 10.1128/mSphere.00776-21 34643418 PMC8513677

[B31] Wick R. R. Judd L. M. Gorrie C. L. Holt K. E. (2017). Unicycler: resolving bacterial genome assemblies from short and long sequencing reads. *PLoS Comput. Biol.* 13:e1005595. 10.1371/journal.pcbi.1005595 28594827 PMC5481147

[B32] Williamson D. A. Sidjabat H. E. Freeman J. T. Roberts S. A. Silvey A. Woodhouse R. (2012). Identification and molecular characterisation of new delhi metallo-β-lactamase-1 (NDM-1)- and NDM-6-producing *Enterobacteriaceae* from new zealand hospitals. *Int. J. Antimicrob. Agents* 39 529–533. 10.1016/j.ijantimicag.2012.02.017 22526013

[B33] Wu W. Feng Y. Tang G. Qiao F. Mcnally A. Zong Z. (2019). NDM metallo-β-lactamases and their bacterial producers in health care settings. *Clin. Microbiol. Rev.* 32 e115–e118. 10.1128/cmr.00115-18 30700432 PMC6431124

[B34] Xanthopoulou K. Urrutikoetxea-Gutiérrez M. Vidal-Garcia M. Diaz De Tuesta Del Arco J. L. Sánchez-Urtaza S. Wille J. (2020). First report of new delhi metallo-β-lactamase-6 (NDM-6) in a clinical *Acinetobacter baumannii* isolate from northern spain. *Front. Microbiol.* 11:589253. 10.3389/fmicb.2020.589253 33240245 PMC7683408

[B35] Yang X. Dong N. Chan E. W. Zhang R. Chen S. (2021). Carbapenem resistance-encoding and virulence-encoding conjugative plasmids in *Klebsiella pneumoniae*. *Trends Microbiol.* 29 65–83. 10.1016/j.tim.2020.04.012 32448764

[B36] Yang X. Liu W. Liu Y. Wang J. Lv L. Chen X. (2014). F33: A-: B-, IncHI2/ST3, and IncI1/ST71 plasmids drive the dissemination of fosA3 and blaCTX-M-55/-14/-65 in *Escherichia coli* from chickens in China. *Front. Microbiol.* 5:688. 10.3389/fmicb.2014.00688 25566207 PMC4267423

[B37] Yong D. Toleman M. A. Giske C. G. Cho H. S. Sundman K. Lee K. (2009). Characterization of a new metallo-beta-lactamase gene, blaNDM-1, and a novel erythromycin esterase gene carried on a unique genetic structure in *Klebsiella pneumoniae* sequence type 14 from India. *Antimicrob. Agents Chemother.* 53 5046–5054. 10.1128/aac.00774-09 19770275 PMC2786356

[B38] Zafer M. M. Hussein A. F. A. Al-Agamy M. H. Radwan H. H. Hamed S. M. (2021). Genomic characterization of extensively drug-resistant NDM-producing *Acinetobacter baumannii* clinical isolates with the emergence of novel blaADC-257. *Front. Microbiol.* 12:736982. 10.3389/fmicb.2021.736982 34880837 PMC8645854

[B39] Zhang R. Liu L. Zhou H. Chan E. W. Li J. Fang Y. (2017). Nationwide surveillance of clinical carbapenem-resistant *Enterobacteriaceae* (CRE) strains in China. *EBioMedicine* 19 98–106. 10.1016/j.ebiom.2017.04.032 28479289 PMC5440625

[B40] Zhang Z. Guo H. Li X. Li W. Yang G. Ni W. (2021). Genetic diversity and characteristics of blaNDM-positive plasmids in *Escherichia coli*. *Front. Microbiol.* 12:729952. 10.3389/fmicb.2021.729952 34867846 PMC8636099

[B41] Zhao Q. Y. Zhu J. H. Cai R. M. Zheng X. R. Zhang L. J. Chang M. X. (2021). IS26 is responsible for the evolution and transmission of blaNDM-harboring plasmids in *Escherichia coli* of poultry origin in china. *mSystems* 6:e0064621. 10.1128/mSystems.00646-21 34254816 PMC8407110

[B42] Zhao Y. Wang L. Zhang Z. Feng J. Kang H. Fang L. (2017). Structural genomics of pNDM-BTR harboring In191 and Tn6360, and other blaNDM-carrying IncN1 plasmids. *Future Microbiol.* 12 1271–1281. 10.2217/fmb-2017-0067 29027482

[B43] Zhu Y. Jia X. Jia P. Li X. Yang Q. (2021). Genetic and phenotypic characterization of the novel metallo-β-lactamase NDM-29 from *Escherichia coli*. *Front. Microbiol.* 12:743981. 10.3389/fmicb.2021.743981 34659178 PMC8511706

[B44] Zou H. Jia X. Liu H. Li S. Wu X. Huang S. (2020). Emergence of NDM-5-producing *Escherichia coli* in a teaching hospital in chongqing, China: IncF-Type plasmids may contribute to the prevalence of blaNDM-5. *Front. Microbiol.* 11:334. 10.3389/fmicb.2020.00334 32210935 PMC7069339

