# Supplementary material for: Emergence of a Carbapenem-Resistant Klebsiella pneumoniae Isolate Co-harbouring Dual blaNDM– 6-Carrying Plasmids in China
Source: Front Microbiol. 2022 May 18;13:900831. doi: 10.3389/fmicb.2022.900831 (PMC9158518; doi:10.3389/fmicb.2022.900831)
Supplement: Supplementary file 1 [file Table_1.DOCX]

Table S1. The primers used for detection of carbapenemase genes.

| Genes | Forward primer (5’-3’) | Reverse primer (5’-3’) |
| --- | --- | --- |
| *bla*_KPC_ | TGTCACTGTATCGCCGTCTAG | TTACTGCCCGTTGACGCCCAATCC |
| *bla*_OXA-48_ | TTGGTGGCATCGATTATCGG | GAGCACTTCTTTTGTGATGGC |
| *bla*_IMP_ | GGAATAGAGTGGCTTAATTCTC | CCAAACCACTACGTTATC |
| *bla*_NDM_ | TGCCCAATATTATGCACCCGG | CGAAACCCGGCATGTCGAGA |
| *bla*_VIM_ | GATGGTGTTTGGTCGCATA | CGAATGCGCAGCACCAG |
